# Supplementary material for: Mathematical model explains differences in Omicron and Delta SARS-CoV-2 dynamics in Caco-2 and Calu-3 cells
Source: PeerJ. 2024 Mar 27;12:e16964. doi: 10.7717/peerj.16964 (PMC10981414; doi:10.7717/peerj.16964)
Supplement: Supplemental Information 1 [file peerj-12-16964-s001.pdf]

SUPPORTING FIGURES

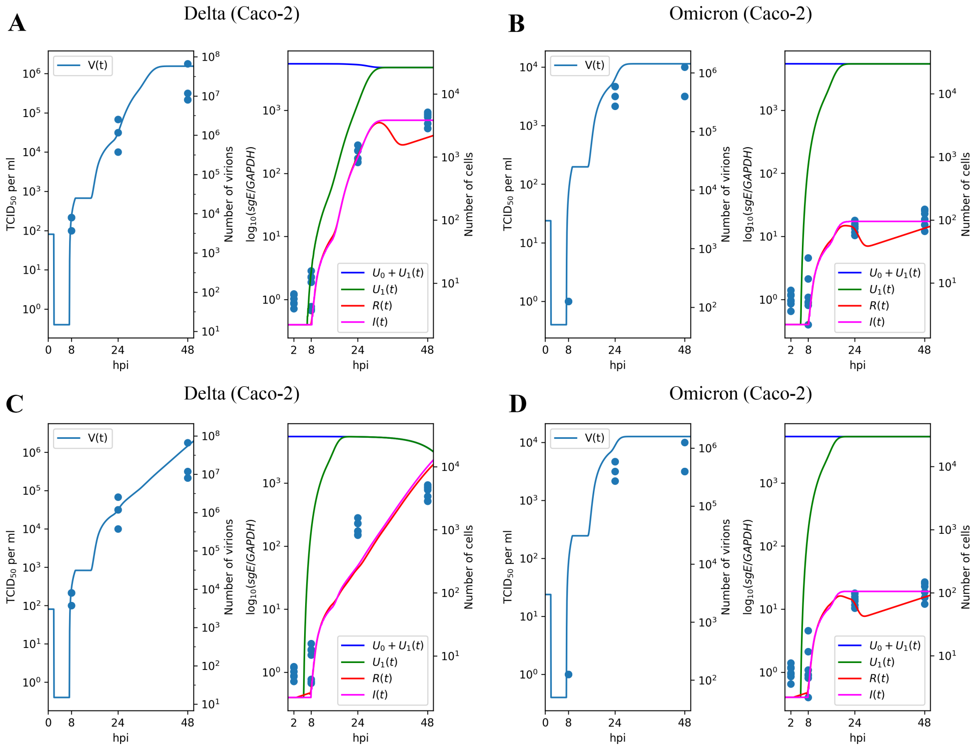

Figure S1. Results of parameter fitting for Caco-2 cell line. The top row shows the results for Delta (A) and Omicron (B) variants with a single variant-specific parameter (the cytokine generation rate p_c). The bottom row shows the results for Delta (C) and Omicron (D) variants with a single variant-specific parameter (the infectivity reduction rate k). The left parts of panels (A), (B), (C), and (D) show $TCID_{50}$ assay values (infectious virus titers); the right parts show RT-qPCR results for subgenomic envelope viral RNA (*sgE*), normalized by the human housekeeping gene *GAPDH*. The following parameter values were inferred for the top row: $\beta = 9.23736 \cdot 10^{-9}$, $k = 3.46004 \cdot 10^{7}$, $p_c = 12389.2$ (Delta), $p_c = 573035$ (Omicron). The following parameter values were inferred for the bottom row: $\beta = 1.14462 \cdot 10^{-8}$, $k = 10.001$ (Delta), $k = 1. \cdot 10^{9}$ (Omicron), $p_c = 626586$.

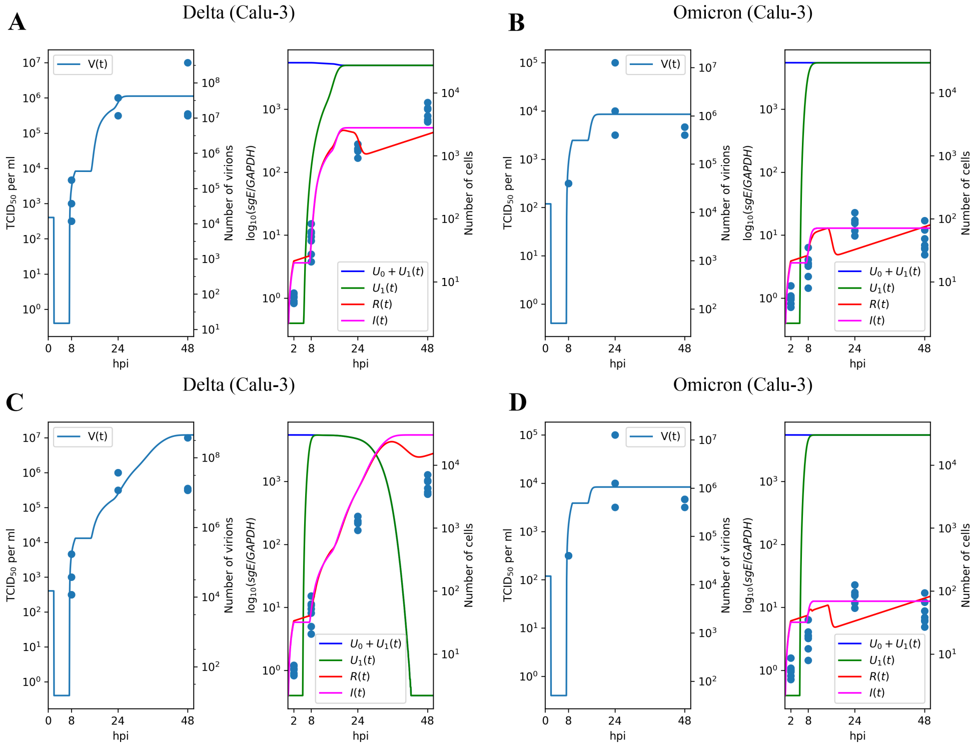

Figure S2. Results of parameter fitting for Calu-3 cell line. The top row shows the results for Delta (A) and Omicron (B) variants with a single variant-specific parameter (the cytokine generation rate p_c). The bottom row shows the results for Delta (C) and Omicron (D) variants with a single variant-specific parameter (the infectivity reduction rate k). The left parts of panels (A), (B), (C), and (D) show TCID$_{50}$ assay values (infectious virus titers); the right parts show RT-qPCR results for subgenomic envelope viral RNA (*sgE*), normalized by the human housekeeping gene *GAPDH*. The following parameter values were inferred for the top row: $\beta = 2.29588 \cdot 10^{-8}$, $k = 9.48894 \cdot 10^{8}$, $p_c = 40612.4$ (Delta), $p_c = 1.86387 \cdot 10^{6}$ (Omicron). The following parameter values were inferred for the bottom row: $\beta = 3.61456 \cdot 10^{-8}$, $k = 10.0223$ (Delta), $k = 1. \cdot 10^{9}$ (Omicron), $p_c = 2.12825 \cdot 10^{6}$.
